# B-cell lymphoma 6 alleviates nonalcoholic fatty liver disease in mice through suppression of fatty acid transporter CD36

**DOI:** 10.1038/s41419-022-04812-x

**Published:** 2022-04-18

**Authors:** Hao Zhang, Yue Li, Chao Zhang, Kun Huang, Jing Zhao, Sheng Le, Lang Jiang, Hao Liu, Peiwen Yang, Xiaoyue Xiao, Jizhang Yu, Jie Wu, Ping Ye, Jiahong Xia

**Affiliations:** 1 Department of Thoracic Surgery, Union Hospital, Tongji Medical College, Huazhong University of Science and Technology, Wuhan, China; 2 Department of Cardiology, the First Affiliated Hospital of Zhengzhou University, Zhengzhou, China; 3 Institute of Pathology, Tongji Hospital, Tongji Medical College, Huazhong University of Science and Technology, Wuhan, China; 4 Department of Cardiology, Union Hospital, Tongji Medical College, Huazhong University of Science and Technology, Wuhan, China; 5 Department of Cardiology, The Central Hospital of Wuhan, Tongji Medical College, Huazhong University of Science and Technology, Wuhan, China; 6 Department of Cardiovascular Surgery, Union Hospital, Tongji Medical College, Huazhong University of Science and Technology, Wuhan, China

**Keywords:** Experimental models of disease, Transcriptional regulatory elements

## Abstract

Nonalcoholic fatty liver disease (NAFLD) is an ubiquitous disease that exists across a wide spectrum ranging from steatosis, steatohepatitis, advanced fibrosis, and liver cirrhosis. Hallmarks of NAFLD are lipid accumulation, insulin resistance, and chronic low-grade inflammation. However, there currently are no medications approved for NAFLD. B-cell lymphoma 6 (BCL6) is a transcriptional inhibitor that is vital for germinal center B-cell formation. Our study identified BCL6 as a critical modulator of hepatic lipid metabolism and appears to contribute to the initiation and progression of NAFLD. In our research, we induced hepatic BCL6 overexpression using adeno-associated virus (AAV), as well as conditional liver-specific BCL6 knockout mice (BCL6-CKO). With these models, we noted that BCL6 overexpression improved insulin resistance and hepatic steatosis in mice models maintained on a HFD diet. Conversely, these parameters worsened in the livers of mice with downregulated BCL6 levels. Mechanistically, the translocase fatty acid CD36 was determined to be a transcriptional target of BCL6 that influences its role in hepatic steatosis. BCL6 bound directly to the CD36 promoter region, restraining CD36 transcription under physiological conditions. We conclude that the hepatocyte BCL6 inhibits the NAFLD progression in mice, including deranged lipid accumulation and glucose metabolism, through a CD36-dependent manner. These results indicate that BCL6 may potentially be targeted in NAFLD treatment.

## Introduction

Obesity rates around the world have reached staggering numbers. Consequently, the most common liver disease worldwide has been found to be nonalcoholic fatty liver disease (NAFLD), a condition marked by an aberrant accumulation of triglycerides (TG) in the liver cells [1, 2]. NAFLD arises from altered hepatic lipid metabolism, which encompasses an accumulation of TG-rich liver droplets in hepatic cells, inefficient triglyceride disposaling, and increased uptake and synthesis of fatty acids (FA). NAFLD progresses from an entity known as nonalcoholic steatohepatitis (NASH) to liver cirrhosis due to chronic hepatic inflammation which causes liver damage and culminates in fibrosis [3]. Moreover, NAFLD patients often have other risk factors for metabolic diseases such as hypertension, dyslipidemia, and insulin resistance [1, 2]. As there is currently no curative mediation for NAFLD, its pathogenesis has been scrutinized in efforts to determine potentially significant molecular targets.

The BCL6 (B-cell lymphoma 6) is a sequence-specific transcription repressor and proto-oncogene that is the cornerstone in lymphoid neoplasms and facilitates innate and adaptive components of the immune system [4, 5]. Despite the ubiquity of BCL6, this protein has been best characterized in the immune system. Previous research showed that inhibition of BCL6 by FX1 in macrophages alleviate the inflammatory response (FX1) [6]. Recently, research has suggested that BCL6 is also involved in the regulation of lipid metabolism. Compared to WT mice, mice with BCL6 deficiency demonstrated significantly reduced adipose tissue mass [7]. In adipocyte-specific knockout mice, BCL6 suppression increased insulin sensitivity and the size and quality of adipocyte tissue in the groin rather than around the groin [8]. BCL6 regulates the dormancy of brown fat cells to maintain thermogenesis and health [9]. BCL6 is enriched in the fed state and converges genome-wide with PPARα to effectively inhibit the induction of fasting transcription of genes [10].

However, how BCL6 regulates lipid metabolism in the context of NAFLD remains poorly understood. Considering that BCL6 is widely involved in the progression of NAFLD as well as the fact that fatty liver previously demonstrates markedly reduced BCL6 expression, we speculate that aberrant BCL6 expression may have an important influence on NAFLD.

## Results

### Hepatic BCL6 expression is decreased in genetic- and high-fat diet (HFD)-induced obese mice and NAFLD patients

**Fig. 1:**
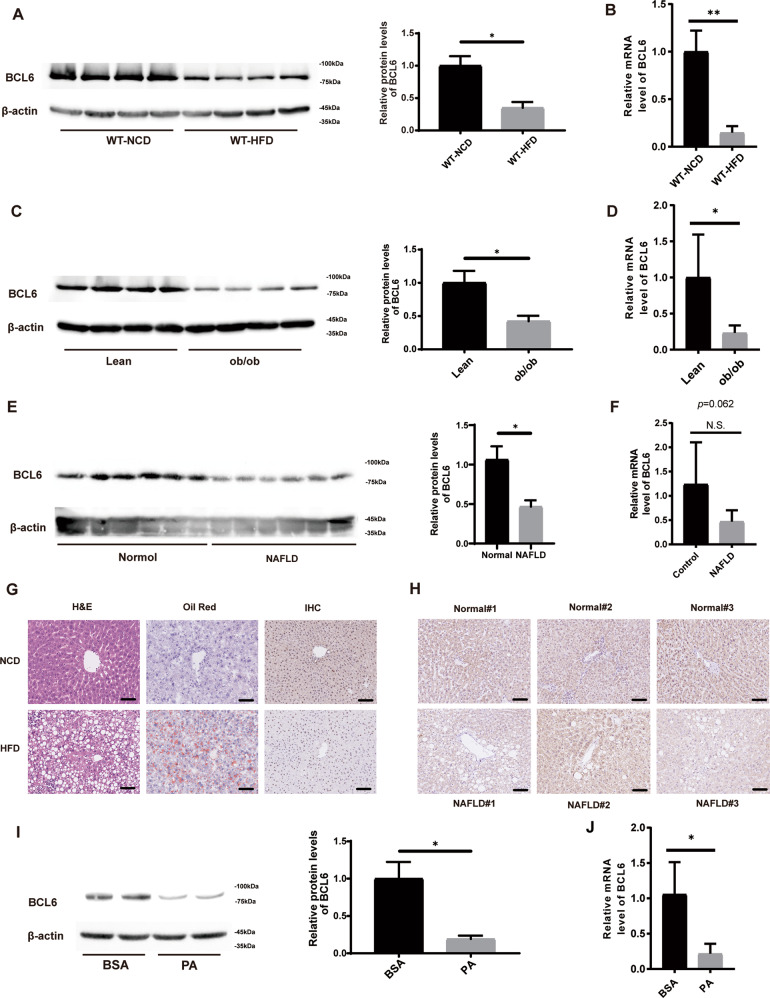
Hepatic BCL6 expression is decreased in genetic- and high-fat diet (HFD)-induced obese mice and NAFLD patients. **A** Protein expression of BCL6 in livers of WT mice fed the NCD or HFD for 16 weeks (*n* = 4). **B** Hepatic mRNA expression of BCL6 of WT mice fed the NCD or HFD for 16 weeks (*n* = 6). mRNA expression of target genes was normalized to that of β-actin. **C** Protein expression of BCL6 in livers of lean mice and genetically obese mice (ob/ob mice) after 8 weeks of NCD (*n* = 4). **D** Hepatic mRNA expression of BCL6 of lean mice and ob/ob mice after 8 weeks of NCD (*n* = 4). **E** Protein expression of BCL6 in livers from normal subjects (*n* = 6) and NAFLD patients (*n* = 6). **F** Relative mRNA levels of BCL6 in livers from normal subjects (*n* = 6) and NAFLD patients (*n* = 6). **G** Representative images of H&E, Oil red O staining, hepatic BCL6 expression by immunohistochemistry of liver tissues from the 16-week-old NCD or HFD mice. Scale bar, 100 μm. **H** Representative immunohistochemistry staining of BCL6 in liver sections from normal subjects and NAFLD patients. Scale bar, 100 μm. **I** BCL6 protein expression in primary hepatocytes, which were isolated and stimulated with PA (0.3 mM) for 24 h. The data represent mean ± SD. **J** mRNA level of BCL6 in primary hepatocytes, which were isolated and stimulated with PA (0.3 mM) for 24 h. mRNA expression of target genes was normalized to that of β-actin. Data represent the mean ± SEM, *P < 0.05, **P < 0.01, ***P < 0.001 and N.S. indicates no significance between the two indicated groups.

BCL6 expression was quantified in liver samples from HFD-induced and ob/ob mice. Both protein and mRNA expression of BCL6 was reduced in HFD-induced mice in contrast to NCD-induced mice (Fig. 1A, B). Similarly, BCL6 expressions were also decreased in ob/ob mice in contrast to their lean counterparts (Fig. 1C, D). These findings correlated to immunohistochemistry results, which also demonstrated decreased BCL6 expression in hepatic cells of HFD-induced obese mice (Fig. 1G). Moreover, we analyzed protein and mRNA expression of BCL6 in the livers of human NAFLD patients. Our results showed that BCL6 protein was decreased in the patients with NAFLD than in the normal subjects (Fig. 1E, F), whereas no significant difference was observed in mRNA expression of BCL6. The decreased expression of BCL6 was confirmed by immunohistochemistry (Fig. 1H). Studies find that palmitate acid (PA) and oleate acid (OA) were the most abundant FAs in liver triglycerides of both NAFLD and normal patients. In order to determine if BCL6 levels fluctuated in lipid-exposed hepatocytes, primary hepatic cells were isolated from wild-type (WT) mice and were exposed to either vehicle, PA FAs, or OA FAs to overload hepatocytes with lipid particles. In contrast to vehicle-treated cells, both protein and mRNA expression of BCL6 in hepatocytes were lowered in response to PA exposure (Fig. 1I, J). Interestingly, OA exposure did not induce the same BCL6 response as seen in PA-stimulated hepatocytes. Both OA and control hepatocytes had similar expressions of BCL6 (Supplement Fig. S1. A, B). These findings suggest that NAFLD pathology is dependent of BCL6 expression.

### Overexpression of BCL6 inhibits hepatic steatosis in HFD-fed obese mice

**Fig. 2:**
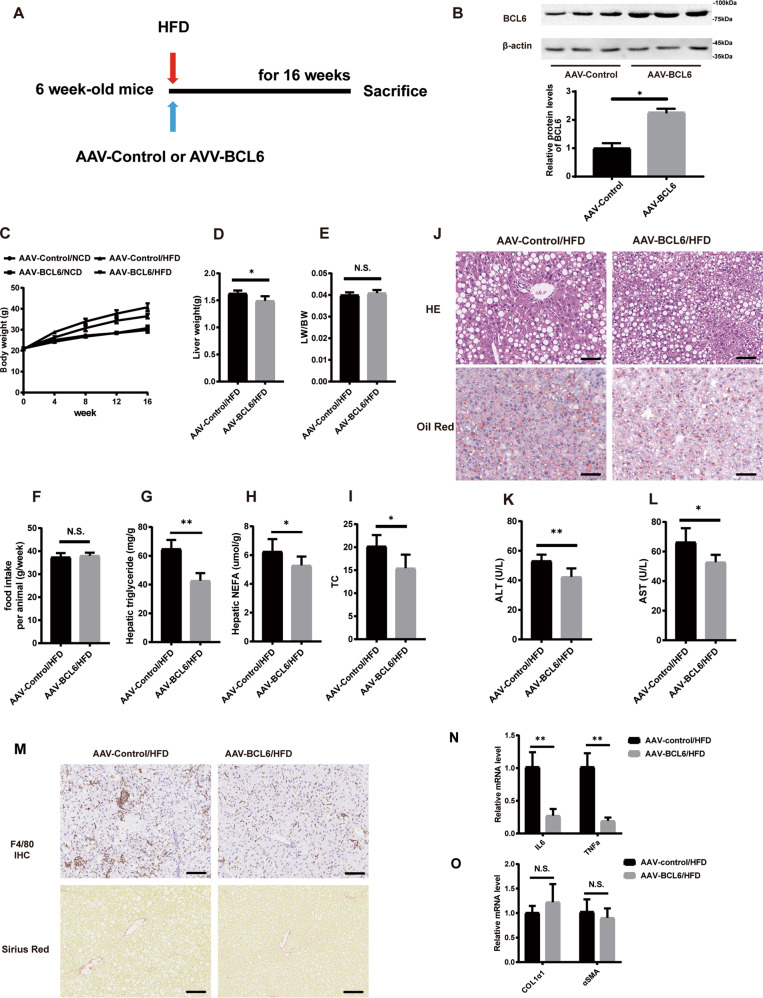
Overexpression of BCL6 alleviates HFD-induced hepatic steatosis. **A** Schedule of BCL6 overexpression. Six-week-old mice were injected with AAV-Control (as a control) or AAV-BCL6 Flag (overexpressing BCL6) via the tail vein. **B** The protein expression of BCL6 was tested by western blot analysis. **C** Bodyweight, **D** liver weight, **E** LW/BW, and **F** food intake of the AAV-Control mice and the AAV-BCL6 mice (*n* = 6/group). **G** Triglyceride, **H** NEFA, **I** total cholesterol in the livers of the AAV-Control mice and the AAV-BCL6 mice fed an HFD for 16 weeks (*n* = 6/group). **J** Representative images of H&E and Oil red O staining of liver tissues from the AAV-Control mice and the AAV-BCL6 mice fed an HFD for 16 weeks. Scale bar, 100 μm. **K** AST and **L** ALT levels in liver tissues from the AAV-Control mice and the AAV-BCL6 mice fed an HFD for 16 weeks (*n* = 6/group). **M** Representative images of immunohistochemistry staining of F4/80 and sirian red staining of liver tissues from two group mice. Scale bar, 100 μm. Hepatic mRNA levels of (N)IL-6 and TNFα, (**O**) COL1α1and αSMA from two group mice fed an HFD for 16 weeks (*n* = 6/group). Data represent the mean ± SEM, *P < 0.05, **P < 0.01,***P < 0.001 and N.S. indicates no significance between the two indicated groups.

To further clarify the effect of BCL6 on lipid metabolismin vivo, we injected hepatocyte-specific BCL6 AAV through the tail of C57BL/6J mice (Fig. 2A). BCL6-AAV inoculation led to BCL6 protein enhancement in the mice livers (Fig. 2B), but not in other examined tissues (data not shown). Control mice (AAV-GFP injected) and AAV-BCL6 injected mice subjected to 16 weeks of HFD demonstrated increased liver and body weights in contrast to their NCD-exposed counterparts. Mice injected with AAV- BCL6 (AAV-BCL6/HFD) demonstrated remarkably slower liver and body weights increase in contrast to AAV- GFP injected mice (AAV-GFP/HFD) (Fig. 2C, D). There were no significant differences between the two mice groups in terms of their food intake and ratio of liver-to-bodyweight (LW/BW) (Fig. 2E, F). Furthermore, H&E and red oil O-stained liver sections staining revealed that hepatic TG, TC, and nonesterified fatty acids (NEFAs) were significantly reduced in the AAV-BCL6/HFD group (Fig. 2G–I for hepatic TG, TC, NEFAs, Fig. 2J for H&E staining and red oil staining). Mice in the AAV-BCL6/HFD group also demonstrated lower alanine aminotransferase (ALT) and aspartate aminotransferase (AST) in contrast to the control mice (Fig. 2K, L). In addition, Mice in the AAV-BCL6/HFD group demonstrated remarkably less inflammatory infiltration in the liver than the control group mice. There were no significant differences in Sirian red staining between the two group mice (Fig. 2M). Similarly, the hepatic mRNA level of IL-6 and TNFα of AAV-BCL6/HFD group mice were significantly lower than control group mice (Fig. 2N). No significant differences were observed in the hepatic mRNA level of COL1α1 and αSMA in two group mice (Fig. 2O). Interpreted as a whole, BCL6 overexpression in hepatocytes alleviates in vivo hepatic steatosis and inflammatory infiltration, while barely affects liver fibrosis.

### Hepatocyte-specific BCL6 deficiency worsens HFD-stimulated obesity and hepatic steatosis

**Fig. 3:**
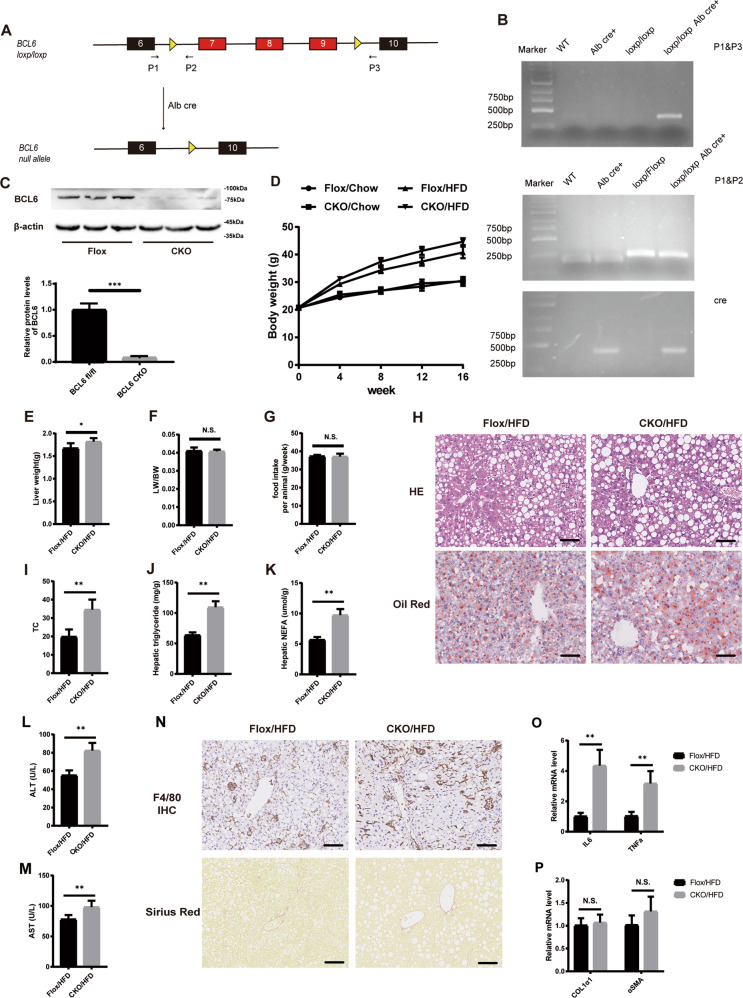
BCL6 knockout aggravates HFD-induced hepatic steatosis. **A** Schematic image of the generation of a liver-specific BCL6-CKO mouse (BCL6-CKO) strain. **B** Mouse genotyping was confirmed by PCR. **C** Protein expression of BCL6 in the liver of flox and CKO mice. **D** Bodyweight, (**E**) liver weight, (**F**) LW/BW, and (**G**) food intake of the BCL6-CKO and BCL6-flox mice at 16 weeks post-HFD administration (*n* = 6/group). **H** Representative images of H&E (upper) and oil red O (bottom) staining of liver tissues from the BCL6-CKO and BCL6-flox mice fed an HFD for 16 weeks. Scale bar, 100 μm. **I** Total cholesterol, **J** triglyceride, **K** NEFA, and **L** ALT and **M** AST levels in the livers from the BCL6-CKO and BCL6-flox mice fed an HFD for 16 weeks. (*n* = 6/group). **N** Representative images of immunohistochemistry staining of F4/80 and Sirian red staining of liver tissues from two group mice. Scale bar, 100 μm. Hepatic mRNA levels of (**O**) IL-6 and TNFα, (**P**) COL1α1and αSMA from two group mice fed an HFD for 16 weeks (*n* = 6/group). Data represent the mean ± SEM, *P < 0.05, **P < 0.01, ***P < 0.001 and N.S. indicates no significance between the two indicated groups.

The BCL6-CKO offspring were then used to examine the effects of BCL6 deficiency in the liver (Fig. 3A–C). BCL6 expression was nearly no change in other organs in BCL6-CKO mice (Supplementary Fig. S2). The BCL6-CKO mice and their age-matched control littermates were exposed for 16 weeks to either HFD or NCD. The bodyweight, liver weight, ratio of liver-to-bodyweight (LW/BW), and food intake were gradually increased in the BCL6-flox control group of mice, whereas the increase of bodyweight and liver weight was more evident in the CKO mice (Fig. 3D–G). Moreover, H&E-stained and oil red O–stained liver sections (Fig. 3H) demonstrated markedly increased hepatic TG, TC, and NEFA levels in HFD-fed in BCL6-CKO mice (Fig. 3I–K). In addition, a significant increase in serum level of ALT and AST were observed in HFD-fed BCL6-CKO mice compared with HFD-fed BCL6-Flox mice (Fig. 3L, M). Furthermore, HFD-fed BCL6-CKO mice demonstrated remarkably aggravated inflammatory infiltration in the liver than BCL6-flox control group mice. There were no significant differences in Sirian red staining between the two group mice (Fig. 3N). Similarly, the hepatic mRNA level of IL-6 and TNFα of BCL6-CKO /HFD group mice were significantly higher than control group mice. No significant differences was observed in hepatic mRNA level of COL1α1 and αSMA in two group mice(Fig. 3O, P). Our findings indicate that BCL6 hepatocyte deletion worsens in vivo hepatic steatosis and inflammatory infiltration, while barely affects liver fibrosis.

### Hepatic BCL6 regulates HFD-triggered insulin resistance

**Fig. 4:**
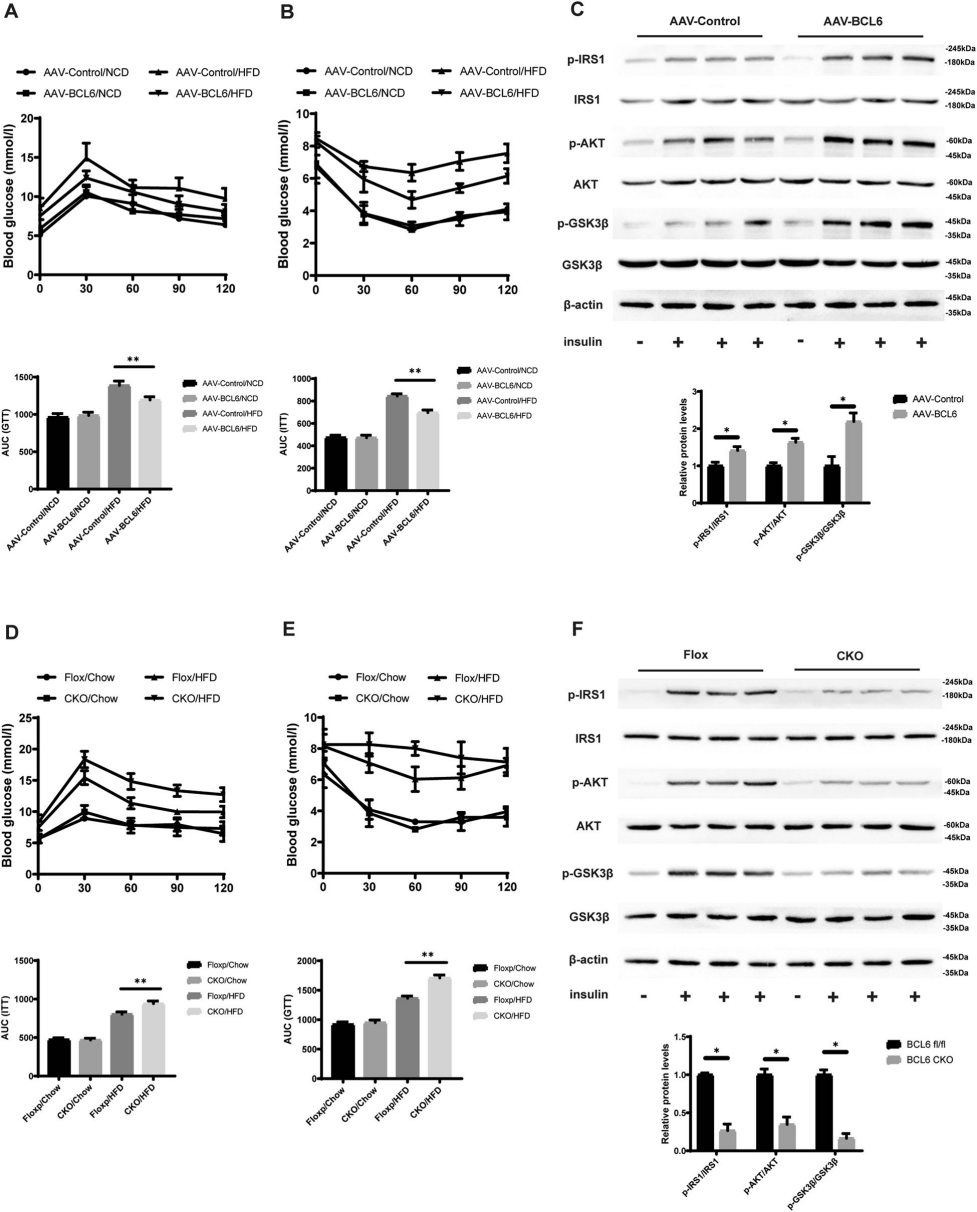
Insulin resistance induced by the HFD can be mitigated by hepatocyte-specific BCL6 overexpression and exacerbated by hepatocyte-specific BCL6 knockout. **A** Intraperitoneal glucose tolerance tests (GTTs; 1 g/kg) and (**B**) intraperitoneal insulin tolerance tests (ITTs; 0.75 U/kg) were performed on the AAV-Control mice and the AAV-BCL6 mice at the 16th week of food administration. The corresponding area under the curve (AUC) of the blood glucose level was calculated (*n* = 6/group). **C** Representative western blot analysis (*n* = 3 western blots for each band) of phosphorylated (p-) and total IRS1, AKT, and GSK3β expression in the livers of the AAV-Control mice and the AAV-BCL6 mice fed an HFD for 16 weeks that received insulin treatment (*n* = 2 mice in each group without insulin injection; *n* = 4 mice in each group with insulin injection). **D** Intraperitoneal glucose tolerance tests (GTTs; 1 g/kg) and (**E**) intraperitoneal insulin tolerance tests (ITTs; 0.75 U/kg) were performed on the BCL6-CKO and BCL6-flox mice at the 16th week of food administration. The corresponding area under the curve (AUC) of the blood glucose level was calculated (*n* = 6/group). **F** Representative western blot analysis (*n* = 3 western blots for each band) of phosphorylated (p-) and total IRS1, AKT, and GSK3β expression in the livers of the BCL6-CKO and BCL6-flox mice fed an HFD for 16 weeks that received insulin treatment (*n* = 2 mice in each group without insulin injection; *n* = 4 mice in each group with insulin injection). Data represent the mean ± SEM, *P < 0.05, **P < 0.01, ***P < 0.001 and n.s. indicates no significance between the two indicated groups.

To determine how glucose metabolism is affected by BCL6 expression, an insulin tolerance test (ITT assay) and a glucose tolerance test (GTT assay) were conducted in mice inoculated with AAV- BCL6. AAV- BCL6 injected mice were noted to have remarkably lower serum glucose profiles as indicated by the area under the curves of the ITT and GTT assays, suggesting the BCL6 exerts a positive effect on insulin sensitivity and glucose tolerance (Fig. 4A, B). Consistently, higher levels of key insulin signaling molecules were found to be phosphorylated in the livers of AAV-BCL6 inoculated mice in contrast to AAV-GFP inoculated mice. Molecules examined included glycogen synthase kinase 3 beta (GSK3β), AKT, and insulin receptor substrate 1 (IRS1) (Fig. 4C). Identical tests were conducted in BCL6-CKO mice. Conversely, BCL6-CKO mice demonstrated insulin resistance and had poorer glucose tolerance (Fig. 4D, E). Correspondingly, BCL6-CKO mice had lower phosphorylated levels of IRS1, AKT, and GSK3β in contrast to control mice (Fig. 4F). Furthermore, we explored insulin signaling in vitro using LO2 cells, which respond to insulin or PA stimulation, upon manipulation of BCL6 expression via adenovirus containing BCL6-coding sequence or BCL6 shRNA (Supplementary Fig. S3A, S3B). Consistent with the in vivo test, the in vitro results indicate that phosphorylated levels of IRS1, AKT were higher upon BCL6 overexpression, while lower in absence of BCL6. These findings suggest that BCL6 has a considerable role in insulin resistance.

### Overexpression of BCL6 inhibits CD36 expression both in vivo and in vitro

**Fig. 5:**
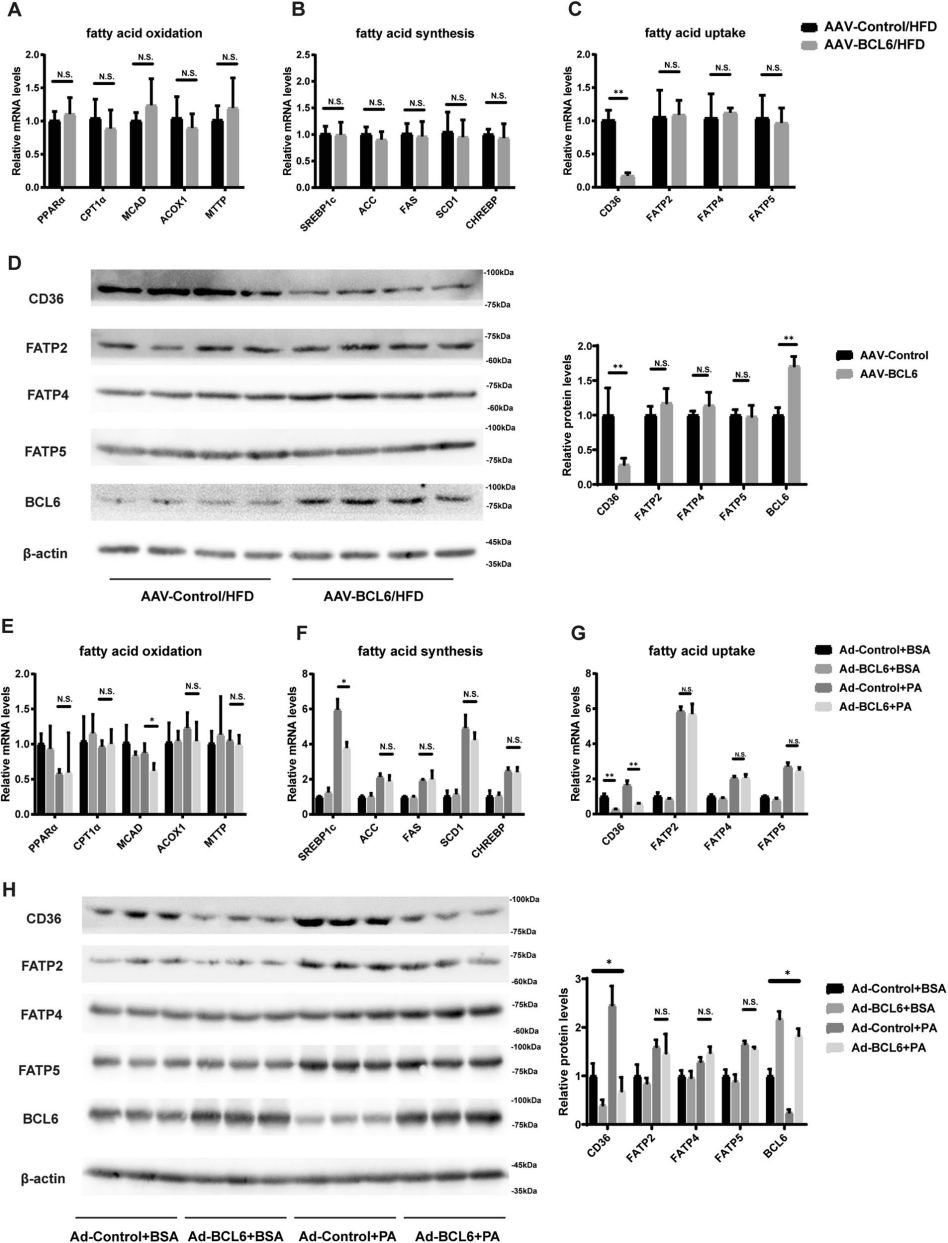
Overexpression of BCL6 hampers CD36 expression both in vivo and in vitro. Relative mRNA levels of genes related to (**A**) FFA oxidation, (**B**) synthesis, and (**C**) uptake in the livers of AAV-Control mice and the AAV-BCL6 mice fed an HFD for 16 weeks. **D** Protein level of BCL6 and genes related to FFA uptake in the livers of mice. Relative mRNA levels of gene related to (**E**) FFA oxidation, (**F**) synthesis and **G** uptake in the livers of AAV-Control mice and the AAV-BCL6 mice fed an HFD for 16 weeks. **D** Protein level of BCL6 and genes related to FFA uptake in the livers of mice. Relative mRNA levels of gene related to (**E**) FFA oxidation, (**F**) synthesis, and (**G**) uptake in the LO2 cells stimulated with PA (0.3 mM) for 24 h after transfected with Ad-GFP or Ad-BCL6 for 48 h. *n* = 3 in each group. **H** Protein level of BCL6 and genes related to FFA uptake in the LO2 cells. Data represent the mean ± SEM, *P < 0.05, **P < 0.01, ***P < 0.001 and n.s. indicates no significance between the two indicated groups.

To examine the role of BCL6 in lipid metabolic pathway regulation, selected FA-related metabolism genes were assessed in HFD-fed mice injected with or without AAV-BCL6. Figure 5A–C demonstrates that AAV-BCL6 injected HFD-fed mice did not alter most hepatic mRNA of genes involved in FAO (PPARα, CPT1a, Acox1, MCAD, MTTP), FA synthesis (SREBP1c, ACC, FAS, SCD1, CHREBP), and FA uptake (FATP2, FATP4, FATP5) in contrast to their respective controls. Of all the FA uptake-related genes tested, both protein and mRNA levels of CD36 were notably suppressed in the AAV-BCL6 group in contrast to its control. No significant change was noted in the FATP2, FATP4, and FATP5 groups (Fig. 5D). The in vivo findings were confirmed by repeating the assessments on LO2 cells transfected by adenovirus containing BCL6-coding sequence (ADV-BCL6) or GFP coding sequence (ADV-GFP). BCL6 was successfully overexpressed in LO2 cells shown in Fig. 5H. These cells were then exposed to PA or BSA prior to the assessment of mRNA and protein levels of the relevant FA metabolism-associated genes. Lipid deposition was alleviated in PA-stimulated LO2 cells after BCL6 overexpression (Supplementary Fig. S4A). However, in line with the in vivo results, we did not observe obvious effects of BCL6 overexpression on the mRNA levels of genes related to FAO (PPARα, CPT1a, Acox1, MCAD, MTTP), FA synthesis (ACC, FAS, SCD1, CHREBP, except SREBP1c), and FA uptake (FATP2, FATP4, FATP5) (Fig. 5E–G). The change in protein expression of genes involved in FA uptake (CD36, FATP2, FATP4, FATP5) was in accordance to the changes observed in their mRNA levels (Fig. 5H). Based on these findings, we postulate that BCL6 overexpression in hepatic cells predominantly induces hepatic CD36 downregulation while not influencing other FA metabolism-related genes.

### Downregulation of BCL6 promotes CD36 expression both in vivo and in vitro

**Fig. 6:**
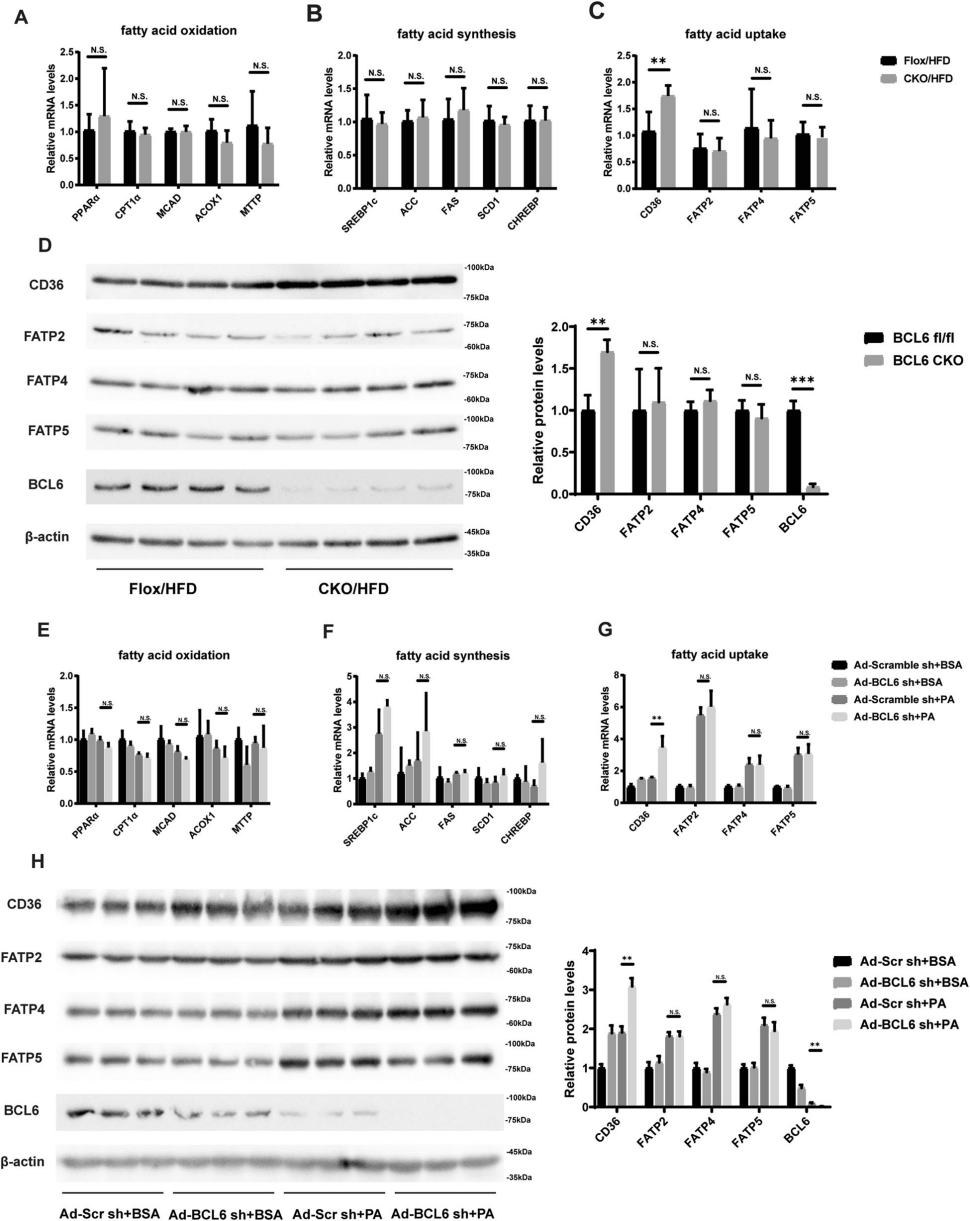
BCL6 knockout promotes CD36 expression both in vivo and in vitro. Relative mRNA levels of genes related to (**A**) FFA oxidation, (**B**) synthesis, and (**C**) uptake in the livers of BCL6-flox and BCL6-CKO mice fed an HFD for 16 weeks. **D** Protein level of BCL6 and genes related to FFA uptake in the livers of mice. Relative mRNA levels of gene related to (**E**) FFA oxidation, (**F**) synthesis, and (**G**) uptake in the livers of BCL6-flox and BCL6-CKO mice fed an HFD for 16 weeks. **D** Protein level of BCL6 and genes related to FFA uptake in the livers of mice. Relative mRNA levels of gene related to (**E**) FFA oxidation, (**F**) synthesis, and (**G**) uptake in the LO2 cells stimulated with PA (0.3 mM) for 24 h after transfected with Ad-Scr sh (as a control) or Ad-BCL6 sh for 48 h. *n* = 3 in each group. **H** Protein level of BCL6 and genes related to FFA uptake in the LO2 cells. Data represent the mean ± SEM, *P < 0.05, **P < 0.01, ***P < 0.001 and n.s. indicates no significance between the two indicated groups.

We next investigated how suppressing BCL6 affected FAO, FA uptake, and FA synthesis. 16 weeks of HFD diets were given to BCL6-CKO mice and their age-matched control littermates for subsequent evaluation of relevant FA metabolism-related genes. As shown in Fig. 6A–C, HFD-fed BCL6-CKO mice did not demonstrate any difference in mRNA expression of genes involved in FAO (PPARα, CPT1a, Acox1, MCAD, MTTP), FA synthesis (SREBP1c, ACC, FAS, SCD1, CHREBP), and FA uptake (FATP2, FATP4, FATP5) in the hepatic tissues in contrast to their controls. Interestingly, BCL6 knockout considerably increased the mRNA level of CD36 in the liver. There was also an obvious increase in CD36 protein levels in HFD-fed BCL6-CKO mice (Fig. 6D). The in vivo findings were confirmed by transfecting LO2 cells with adenovirus containing BCL6 shRNA or scrambled shRNA. As shown in Fig. 6H, BCL6 was successfully knocked down in LO2 cells. These cells were then exposed to PA or BSA prior to the assessment of mRNA and protein levels of the relevant FA metabolism-associated genes. Lipid deposition was aggravated in PA-stimulated LO2 cells after BCL6 knockdown (Supplementary Fig. S4B). Similar to the in vivo results, we did not observe obvious effects of BCL6 knockdown on the mRNA levels of genes related to FAO (PPARα, CPT1a, Acox1, MCAD, MTTP), FA synthesis (ACC, FAS, SCD1, CHREBP, except SREBP1c), and FA uptake (FATP2, FATP4, FATP5) (Fig. 6E–G). Similar patterns in protein expressions of the FA uptake(CD36, FATP2, FATP4, FATP5)-related genes were observed (Fig. 6H). We further carried out RNA-sequencing analysis on the livers of wild-type mice and BCL6 knockout mice in order to study the impact of BCL6 on hepatic lipid metabolism. (Supplementary Fig. S6). Special attention was paid to molecules related to FA transport. RNA sequencing found that CD36 significantly upregulated in BCL6-knockout mice in accordance with Fig. 6A–D. These findings suggest that BCL6 deficiency in hepatic cells induces hepatic CD36 gene upregulation alone and has no effect on other genes related to FA synthesis, uptake, and oxidation.

### BCL6 inhibits CD36 expression by directly regulating CD36 promoter

**Fig. 7:**
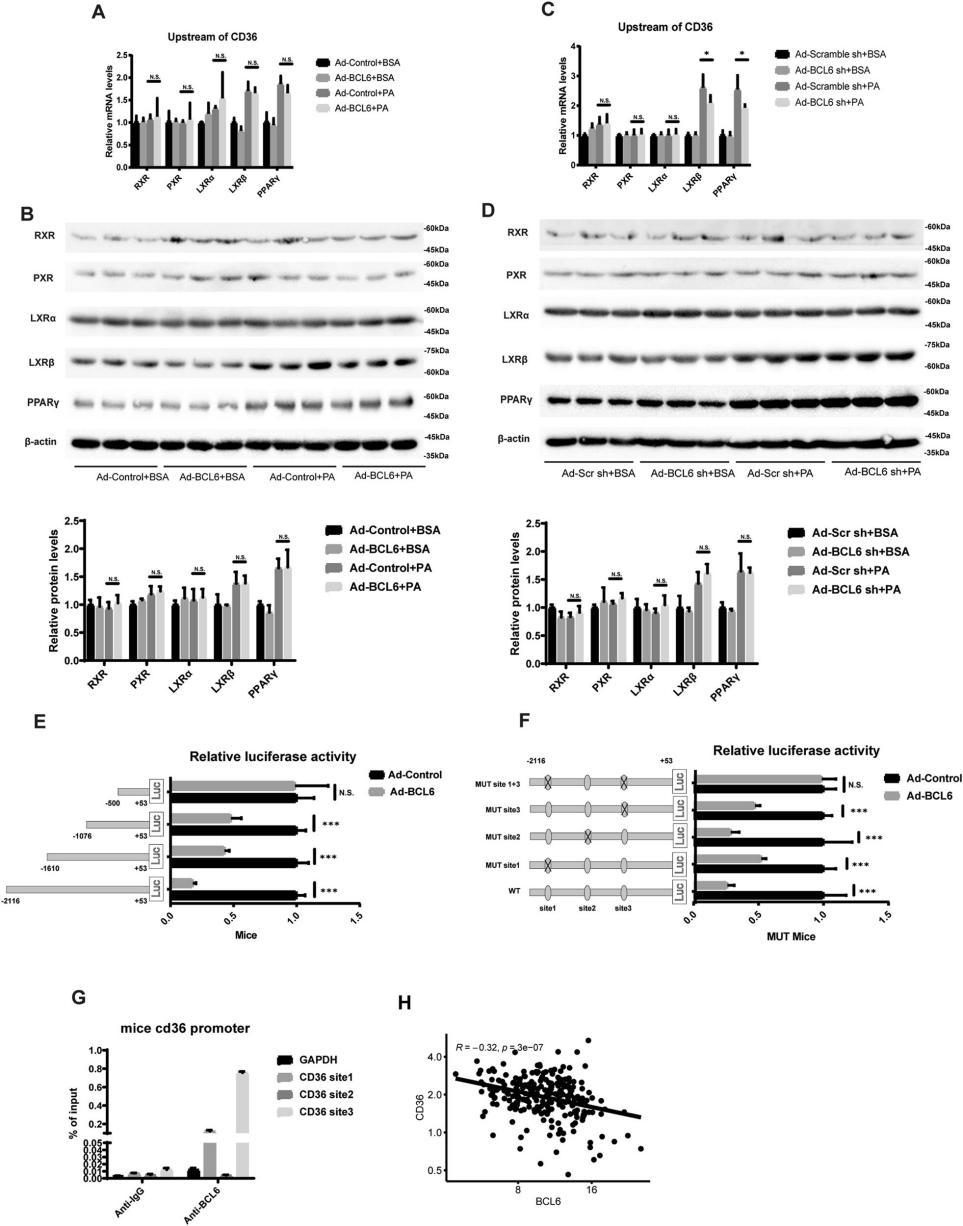
BCL6 regulates CD36 expression. **A** Relative mRNA and (**B**) protein levels of RXR, PXR, LXRα, LXRβ, and PPARγ in the LO2 cells stimulated with PA (0.3 mM) for 24 h after transfected with Ad-Control or Ad-BCL6 for 48 h. *n* = 3 in each group. **C** Relative mRNA levels and (**D**) protein of RXR, PXR, LXRα, LXRβ, and PPARγ in the LO2 cells stimulated with PA (0.3 mM) for 24 h after transfected with Ad-Scr sh (as a control) or Ad-BCL6 sh for 48 h. *n* = 3 in each group. **E** Relative luciferase activity of CD36 promoters of different lengths in primary hepatocytes of mice after BCL6 overexpression or not. **F** Relative luciferase activity of CD36 promoters after mutation of different sites in primary hepatocytes of mice after BCL6 overexpression or not. **G** Quantitative ChIP was performed in the LO2 cells using antibodies for BCL6 or IgG control to enrich for possible BCL6 binding sites in the CD36 loci. The *y* axis represents fold enrichment of binding versus input, as compared with IgG control. **H** Pearson *r* and *P* values for normalized BCL6 mRNA levels versus normalized CD36 mRNA levels in human livers (*n* = 243). Data represent the mean ± SEM, *P < 0.05, **P < 0.01, ***P < 0.001 and n.s. indicates no significance between the two indicated groups.

CD36 has been documented to be involved in the regulation of its own expression by modulating a variety of transcription factors, such as RXR, PXR, LXRα, LXRβ, and PPARγ. We then sought to determine if BCL6 directly or indirectly regulated CD36. This problem was clarified by stimulating primary mouse hepatocytes with PA and artificially altering the BCL6 levels prior to studying specific gene expression using real-time PCR and western blotting. We found that BCL6 exerted no effect on the gene expressions at the mRNA or protein level (Fig. 7A–D). Using the database http://jaspar.genereg.net/, we first predicted candidate CD36 mice promoter sites, created variations of these sites, and transfected them into primary mice hepatocytes. BCL6 overexpression inhibited the activity of both −1610 and −1076 length CD36 promoter, but to a weaker degree than the promoter of −2116 length. There was no observed effect in the activity of the promoter of −500 (Fig. 7E). This suggests that the active sites of BCL6 on CD36 exist between −1610–−2116 and −500–−1076. Figure 7F demonstrates the predicted binding sites. We further studied the effect of BCL6 on the reported gene activity by mutating site1 (−1645), site 2 (−1558), site 3 (−899), and site 1 + 3 (−1645, −899). BCL6 was then further confirmed to be either on site1 and site3. Accordingly, various ChIP primers specific to the postulated binding sites of BCL6 were created, which also found that BCL6 potentially bound to SITE1 and SITE3 (Fig. 7G). In JAPAR (http://jaspar.genereg.net/), we predicted that there was a possible binding site (−799) of BCL6 in the human CD36 promoter, and constructed reporter genes with different lengths and binding site mutations. We then studied the effect of BCL6 on CD36 promoter activity in the LO2 liver cell line, and found that BCL6 could affect CD36 expression by binding to the CD36 promoter region (a possible binding site is −799) (Supplementary Fig. S5A). Additional ChIP experiments also confirmed that BCL6 could bind to the CD36 promoter (Supplementary Fig. S5B). Moreover, Pearson correlation analysis showed that mRNA levels of BCL6 and CD36 are negatively and significantly correlated in the human livers according to statistical results of non-hepatocellular liver samples from GSE25097 of the GEO data repository (Fig. 7H). Thus, our results suggest that BCL6 inhibits CD36 expression by targeting CD36 promoter.

### CD36 mediates the effect of BCL6 on hepatic steatosis both in vivo and in vitro

**Fig. 8:**
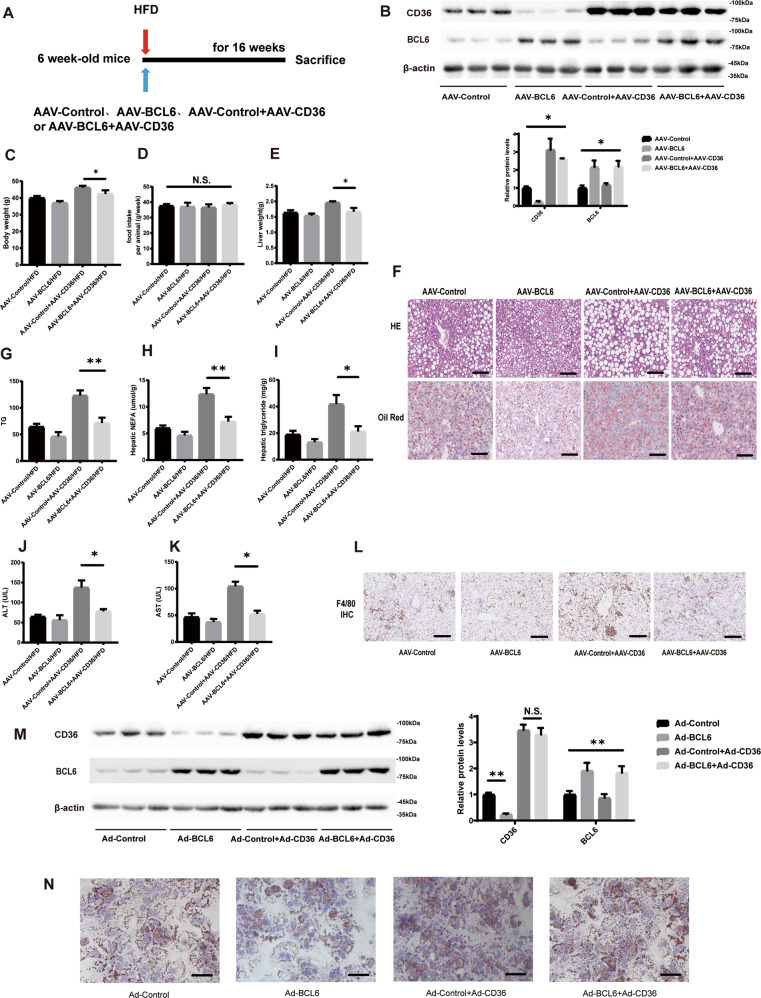
CD36 reversed the phenotype induced by BCL6 overexpression. **A** A Schedule of manipulating the overexpression of CD36 and BCL6. Six-week-old mice were injected with AAV-Control, AVV-BCL6, AAV-Control + AAV-CD36, or AAV-BCL6 + AAV-CD36 via the tail vein and fed an HFD. **B** Protein level of CD36 and BCL6 in livers of the mice from the indicated groups after being injected with AAV. **C** Bodyweight, (**D**) food intake, and (**E**) LW/BW of the mice from the indicated groups (*n* = 6/group). **F** Representative images of H&E and Oil red O staining of liver tissues from the mice from the indicated groups. Scale bar, 100 μm. **G** Total cholesterol, **H** triglyceride, **I** NEFA, **J** ALT, and (**K**) AST levels in the livers from the mice of indicated groups fed an HFD for 16 weeks. (*n* = 6/group). **L** Representative images of immunohistochemistry staining of F4/80 of liver tissues from four group mice. Scale bar, 100 μm. **M** Protein level of CD36 and BCL6 in LO2 cells after transfected adv-Control, adv-BCL6, adv-Control + adv-CD36, or adv-BCL6 + adv-CD36. **N** Representative images of Oil red O staining of LO2 cells of indicated groups stimulated with PA (0.3 mM) for 24 h. Data represent the mean ± SEM, *P < 0.05, **P < 0.01, ***P < 0.001 and n.s. indicates no significance between the two indicated groups.

In order to confirm the interaction of BCL6 and CD36 in modulating lipid accumulation, C57BL/6 mice were injected by AAV-Control, AAV-BCL6, AAV-CD36, or both AAV-BCL6 and AAV-CD36. Figure 8A demonstrates the procedures and feeding protocols. BCL6 and CD36 were successfully overexpressed in the liver as shown in Fig. 8B. After 16 weeks of HFD treatment, AAV-BCL6 injected mice (AAV-BCL6 /HFD) were noted to have lower liver and body weights in contrast to the AAV-Control injected mice(AAV-Control /HFD). CD36 overexpression reversed these effects in the absence of changes in food intake (Fig. 8C–E). Similarly, H&E, red oil O-staining, examined TG, TC, and NEFAs in liver sections also suggested that BCL6 inhibited lipid accumulation. These findings were also reversed by CD36 overexpression (Fig. 8F–I). Liver function trends were also the same (Fig. 8J, K). In addition, Mice in the AAV-BCL6/HFD group demonstrated remarkably less inflammatory infiltration in the liver than control group mice, while these changes were reversed by CD36 overexpression (Fig. 8L). Moreover, Fig. 8M confirms infection with adenovirus containing GFP, CD36, or BCL6, as well as BCL6 and CD36 in LO2 cells. Consistent with these in vivo results, oil red O-staining in LO2 cells also suggested that CD36 overexpression overrode the effect of BCL6 on lipid accumulation and lipid contents (Fig. 8N).

## Discussion

One of the traits of NAFLD is the imbalance in FA uptake, synthesis, oxidation, and export, culminating in excessive hepatic TG [11]. This study finds transcription factor BCL6 to be a critical key regulator of fatty liver disease. We discovered for the first time a BCL6/CD36 axis that centrally modulates obesity-associated NAFLD. Hepatic BCL6 knockout promoted TG accumulation in the liver of healthy mice, while BCL6 overexpression attenuated the severity of fatty liver in obese mice. Finally, our research shows that BCL6 exerts its effect by inhibiting CD36 transcription. These results indicate that BCL6 functions as a promising target in treating deranged FA metabolism in NAFLD.

The function of BCL6 outside of hematopoietic cells has not been clearly defined. In the liver, previous analyses support the role of BCL6 in competing with STAT5 and in regulating the hepatic response to growth hormone and drug metabolism [12, 13]. Furthermore, a study on BCL6 knockout mice showed that BCL6 plays a role in systemic FA metabolism, however, this analysis was limited to animals with severe and frequently fatal inflammatory diseases [7]. The original characteristics of the Bcl6−/− mice showed varying degrees of stunted growth and poor health within three weeks of birth, and half of them died before the age of 5 weeks [14]. Over 80% of Bcl6−/− mice developed myocarditis, and over 70% of mice suffered from pulmonary vasculitis, with high levels of IL-4, −5, and −13. These cytokines are known to directly affect hepatic metabolism [15, 16]. Therefore, the metabolic phenotype of Bcl6 knockout mice has yet to be clearly elucidated [7]. Although a number of studies imply that BCL6 regulates lipid metabolism, BCL6 has not been studied in relation to intracellular hepatic lipid metabolism.

In contrast to the characteristics of systemic BCL6 knockout mice, we found that hepatocyte-specific BCL6-deficient mice exhibited worse outcomes of HFD-induced obesity and hepatic steatosis. Furthermore, we observed that HFD induces a reduction of BCL6 in the fatty liver of NAFLD mice, which has not been previously characterized. Hiromi Chikada and colleagues reported that BCL6 liver knockout mice exhibited suppressed NASH progression, which was caused by feeding mice 7 weeks of the high-fat diet, which was defined as choline-deficient L-amino acids (CDAHFD) [17]. Sommars et al. previously reported that BCL6 is an effective PPARα antagonist that turns off genes involved in fat burning when mice eat [10]. Despite the differences, these results collectively indicate that BCL6 is closely related to hepatic lipid metabolism. Differences in BCL6 expressions may have arisen as a result of variable experimental conditions used or different hepatic liver disease stages.

Mechanistically, BCL6 transcriptionally inhibits the expression of the CD36 gene. Several metabolic organs, including the liver, expresses the transmembrane glycoprotein CD36. Numerous studies have testified to the central function of this receptor in promoting fatty acid uptake, and have demonstrated increased CD36 expressions in the livers of obese mice and NAFLD patients [18–22]. In addition, upregulation of hepatic CD36 is closely related to insulin resistance and hepatic fatty content in patients with NASH [23]. Nevertheless, the physiological and pathophysiological regulators of CD36 expression still need to be largely explored. CD36 has been shown to be the common target of LXR, the pregnan X receptor (PXR) and PPARγ at the transcriptional level [24]. However, PXR, LXRα, LXRβ, and PPARγ expressions were not affected by overexpression or knockdown of BCL6 (Fig. 7C, D).

Here, we hypothesize that CD36 downregulation by BCL6 may not be reliant on other nuclear receptors. In addition, investigations have demonstrated that hepatic CD36 protein levels are increased by inflammatory stress which works by mTOR activation that improves the translational efficiency of CD36 [25]. Moreover, NASH mice were found to have high levels of palmitoylated CD36, and palmitoylation is a crucial step in enabling liver cell plasma membrane transfer, which promotes FA uptake [20]. Our data further highlights the complexity of CD36 expression and regulation in chronic liver disease progression. There are modulators of CD36 expression and activity that have yet to be clarified, and this area of study require special focus. It should be noted that BCL6 upregulation in hepatocytes was sufficient in inhibiting CD36 gene expression and lipid accumulation in the liver, resulting in an overall improvement in HFD mice insulin resistance. Based on these data, therapy aimed at upregulating BCL6 expression may be significant in treating HFD-induced NAFLD.

There is no doubt that NAFLD is a core component of the metabolic syndrome, supporting the fact that fatty liver disease exerts a significant influence on the occurrence of systemic metabolic disorders [26]. Insulin resistance in originally insulin-sensitive hepatocytes causes marked alterations in glucose and lipid metabolism [27]. This study found that the increased hepatic BCL6 expression sufficiently inhibits CD36 gene expression and subsequent hepatic lipid accumulation, while also exerting a positive effect on insulin resistance in HFD-fed mice. Drugs which enhance BCL6 expression or modify the transcriptional regulation of BCL6 and CD36 represent an effective treatment method for NAFLD and associated metabolic diseases.

## Materials and methods

### Animals

B6.129S (FVB)-Bcl6tm1.1Dent/J(Bcl6fl/fl) were obtained from the Jackson Laboratory (Stock No.: 023727). Alb-Cre mice were obtained from the Model Animal Research Center of Nanjing University. Alb-Cre mice were bred with the Bcl6 fl/fl mice to obtain the Alb-Cre Bcl6Δ mice. Genotyping was performed via PCR using previously published protocols [28–30]. The Animal Use Subcommittee of Tongji Medical College of Huazhong University of Science and Technology approved all animal experimental procedures. A specific-pathogen-free facility equipped 12-h light/12-h dark cycles and free access to food and water were used to house six-week-old C57BL/6J mice.

Six-week-old male mice were randomized into two groups to create diet-induced NAFLD – the first of these groups were fed 16 weeks of normal chow diet (NCD) (4% fat, 78% carbohydrate, and 18% protein; H10010, HFK Bioscience, Beijing, China) while the other group received 16 weeks of HFD (60.9% fat, 21.8% carbohydrate, and 18.3% protein; H10060). NCDGene-edited ob/ob mice provided by HFK Bioscience (Beijing, China) were fed NCD for 8 weeks. After this duration, mice were sacrificed, blood samples were harvested, and livers were dissected for immediate paraformaldehyde fixation or storage for further use with frozen liquid nitrogen.

### Human samples

For analysis of hepatic gene expression in humans, the liver tissues were collected in the sample bank of the Institute of Pathology, Tongji Hospital, Tongji Medical College, Huazhong University of Science and Technology. The human study was approved by the Independent Ethics Committee of Union Hospital, Tongji Medical College, Huazhong University of Science and Technology (approval number: [2021] IEC (0687)). The experiments were conducted according to the principles outlined in the Declaration of Helsinki.

### Cell culture

Procell Biotech (Wuhan, China) provided LO2 cells, which were normal human hepatocyte cell lines. DMEM supplemented 10% fetal bovine serum and 1% penicillin-streptomycin was used to maintain the cells. Primary mice hepatocytes were isolated, purified, and cultures as previously documented [dusp9]. Briefly, 60 mg/kg bodyweight of sodium pentobarbital were used to anesthetize mice via intraperitoneal injections. Mice abdominal cavities were entered and the portal vein was identified. The liver was perfused with 5 ml of liver perfusion solution (D-Hank’s, 0.5 mM EGTA, and 10 mM HEPES) to remove blood. This was followed by an infusion of digestive buffer (D-Hank’s, 5 mM collagen II, and 10 mM HEPES) at 2.5–5 ml per minute until liver color changed and elasticity was lost. The surface membrane of the liver was then removed, pieces of the liver dissected, and filtered with a strainer. DMEM with 10% serum was used to resuspend the hepatocytes. Viable hepatocytes were quantified with Trypan blue staining, with hepatocytes planted on a fibronectin-coated cell culture plate. Palmitate (PA) (0.25 mM; P0500; Sigma-Aldrich, St. Louis, MO, USA) was used to establish an in vitro cell lipid deposition model. Cells were stained for 10 min with 60% oil red O (O1391; Sigma) working solution to identify the amount of lipid accumulation. Intracellular triglyceride levels were detected with a triglyceride colorimetric assay kit (10010303; Cayman) was used to detect intracellular triglyceride levels. All these cells were cultured at 37 °C and 5%CO_2_.

### Western blot

A RIPA lysis buffer with PhosSTOP phosphatase inhibitor (Roche Diagnostics, Barcelona, Spain) and complete protease inhibitor cocktail (Roche)) was used to isolate whole-cell lysates from tissues or cells. Protein concentrations were determined with a BCA protein assay kit (Thermo Scientific, Waltham, MA, USA). PAGE gel protein separation was performed, and the proteins were immunoblotted onto a PVDF membrane (Millipore, Billerica, MA, USA). Membranes were then blocked via exposure to nonfat milk in TBST. Primary antibodies were then used to incubate with the membranes overnight at 4 °C: anti-BCL6 (Proteintech, 21187-1-ap), anti-β-actin (Abcam, ab8226), anti-IRS1 (Cell Signaling Technology, 2382), anti-AKT (Cell Signaling Technology, 4691), anti-GSK3β (Cell Signaling Technology, 9315), anti-p-IRS1 (Millipore, 09-432), anti-p-AKT (Cell Signaling Technology, 4060), anti-p-GSK3β (Cell Signaling Technology, 9322), anti-FATP2 (Proteintech, 14048-1-AP), anti-FATP4(Proteintech, 11013-1-AP), anti-FATP5 (Affinity, DF3845), anti-CD36 (Cell Signaling Technology, 14347), anti-RXR (Cell Signaling Technology, 3085), anti-PXR (Abcam, ab192579), anti-LXRα (Proteintech,14351-1-AP), anti-LXRβ (Cell Signaling Technology, 13519), and anti-PPARγ (Cell Signaling Technology, 2435). This was followed by re-incubation with horseradish peroxidase-conjugated secondary antibody for an hour at room temperature. The ChemiDoc XRS + imaging system (Bio-Rad, Hercules, CA, USA) was used to detect chemiluminescence signals. Full-length uncropped original western blots were put in Supplemental Material.

### Recombinant adenovirus and adeno-associated virus production

Recombinant adenovirus containing BCL6, CD36, BCL6 shRNA (a short hairpin RNA targeting BCL6, mouse BCL6 shRNA, green fluorescent protein (GFP, as a negative control), or scrambled shRNA (as a nonspecific control) were used for in vitro infection of primary hepatocytes. Recombinant adeno-associated virus (AAV) system (type 8), which contained control, scramble shRNA and BCL6 shRNA, or BCL6 and CD36 with a liver-specific promoter (ALB promoter) were injected into the tail veins in order to alter CD36 or BCL6 levels. Mice were injected via the tail vein with 100 μl of virus containing 2 × 10^11^ VG of the AAV8 vector genomes. All adenoviruses and adeno-associated viruses were provided by Obio Technology Corp, Ltd. (Shanghai, China).

### Mouse experiments

After 16 weeks of HFD diets, the mice were subjected to assessment of blood glucose levels, body weights, ITTs, and GTTs. ITTs were carried out by fasting mice for 6 h followed by 0.75 U/kg of intraperitoneally injected insulin. GTTs were carried out by fasting mice for 6 h followed by 1 /kg of intraperitoneally injected glucose. Blood glucose concentrations were assessed at 15, 30, 60, and 120 min via blood glucometer evaluation from samples collected from the tail vein. Mice subjected to 16 weeks of HFD were then subjected to acute tissue insulin signaling tests. Mice were first fasted overnight and anesthetized with intramuscular ketamine/xylazine, followed by portal vein injection of human insulin (0.5 U/kg bodyweight) or vehicle saline. Five minutes later, the livers were collected to determine phosphorylation of IRS1, total IRS1, phosphorylation of AKT, total AKT phosphorylation of GSK3β, and total GSK3β by western blotting.

### Histological analysis

Hematoxylin and eosin staining was done on paraffinized specimens to determine lipid accumulation. Frozen liver sections were subjected to oil red O staining to determine lipid droplets in the liver. For immunohistochemical experiments, paraffin-embedded sections labeled overnight with primary antibodies (Bcl6 monoclonal antibody (66340-1-Ig, 1:1000; Proteintech)) overnight were then incubated with horseradish peroxidase-conjugated secondary antibodies. 3,3′-diaminobenzidine (no. ZLI-9032; Zhongshan Biotech, Beijing, China) was used to visualize the specimens. Specimens were imaged using a light microscope (Olympus, Tokyo, Japan).

### RNA-seq analysis

Primary hepatocytes were isolated from Bcl6 fl/fl or CKO mice. Trizol (Life Technologies) reagent was used to extract total RNA, which was then purified. RNA was quantified and screened for quality with a Bioanalyzer 2100 (Agilent Technologies). Illumina HiSeq X Ten was used to construct stranded RNA-seq libraries for high-throughput sequencing in compliance with the manufacturer-supplied instructions. RNA-seq reads were mapped to the reference genome of Illumina Ensembl genome GRCh37 using HISAT2 version 2.2.9. Mapped reads were summarized for each gene using htseq-count version 0.11.2. The DEGseq version 1.36.1. was used for differentially expressed gene analyses. The *P* value was calculated by the Student’s *t* test.

### Luciferase assays

CD36 promoters of various lengths were amplified using PCR with mouse or human genomic DNA and cloned into the Pgl3.0-Basic vector (Promega, Madison, WI) using the One Step Cloning Kit (C112-02, Vazyme Biotech Ltd., Nanjing, China). The putative binding site of BCL6 of the CD36 promoter was deleted by site-directed mutagenesis using a QuikChange II Kit (Stratagene, La Jolla, CA). Luciferase reporter constructs (homo and mice CD36, wild-type, truncated or mutated) were co-transfected with an internal control plasmid pRL-TK (Renilla luciferase reporter plasmid, Promega) into HEK293T cells. The luciferase activity was determined with Dual-Luciferase Reporter Assay Kit (Promega) according to the manufacturer’s instructions.

### Quantitative real-time PCR

Total RNA was extracted from mouse cells and liver tissues using TRIzol (D9108A, TaKaRa Bio). Reverse transcription of isolated RNA into complementary DNA (cDNA) was performed using the RR037A PrimeScript™ RT reagent Kit (Perfect Real Time) (TaKaRa). SYBR Green (Vazyme) was used to quantify the amplification products on a PRISM 7900 Sequence Detector System (Applied Biosystems, Foster City). β-actin was used as the reference gene against other genes. Primer sequences are available upon request.

### Mouse hepatic lipid analyses and serum assays

Commercial kits were used to assess TG, TC, and NEFAs levels (290-63701 for the TG assay, 294-65801 for the TC assay, and 294-63601 for the NEFA assay; Wako, Osaka, Japan). Liver function tests were assessed using a ADVIA 2400 chemical system analyzer (Siemens, Tarrytown, NY, USA), which quantified serum AST and ALT.

### Chromatin immunoprecipitation (ChIP)

In total, 3 × 10^7^ LO2 or mice primary hepatocyte cells were prepared. Cells were fixed for 10 min with 37% formaldehyde (Sigma, USA) at room temperature. 125 mM glycine was added to prevent cross-linking reactions. Cold PBS was used to rinse the cells twice prior to lysing cells with a lysis buffer (Protease inhibitors, 0.1% w/v SDS, 1% v/v Nonidet P-40, 0.5% w/v deoxycholate, 150 mM NaCl, 50 mM Tris pH 8, 5 mM EDTA). Cells were sonicated with a Covaris S220 AFA Ultrasonicator (Covaris Inc., Woburn, MA, USA) for production fragments measuring less than 400 bp. Sonicated lysates were centrifuged, precleared with ChIP-Grade Protein G Magnetic Beads (CST, USA), and incubated overnight at 4 °C with control IgG antibody (CST, USA) or specific BCL6 antibody (Rabbit mAb #14895 CST, USA, dilution 1:200). ChIP results were optimized with 5 μl of antibody and 10 μg of chromatin (~4 × 10^6^ cells) per immunoprecipitation reaction. Cells and 30 μl ChIP-Grade Protein G Magnetic Beads (CST, USA) were gently rocked at 4 °C for 1 h for immunocomplex retrieval. Beads were rinsed twice with a RIPA buffer with increasing ChIP astringency wash buffers at each step (150 mM NaCl, 250 mM NaCl, 250 mM LiCl), and finally with a TE buffer. Immunocomplexes were eluted with an elution buffer comprising of 1% SDS and 100 mM NaHCO3 s. Cross-linking was reverted by the addition of 300 mM NaCl and incubating the samples for at least 5 h at 65 °C. DNA purification was performed with a PCR purification kit (TsingKe, China). A ChIP product fraction served as template in quantitative polymerase chain reaction (PCR) amplification using the ABI PRISM 7900 Sequence Detector System (Applied Biosystem, Foster City, CA) and in 10 μl real-time PCR reactions using SYBR Green (Vazyme, China). Relative enrichment was assessed with input chromatin standard curves.

The following lists the BCL6 target site primers utilized in this study:

**Table:** 

ChIP h CD36 SITE1 F	GAAGGTTCTTTCCCCAAAGTGC
ChIP h CD36 SITE1 R	TGCAAAAATTTCATGATCACAGCA
ChIP m CD36 SITE1 F	TCAGTCAAGTGAATGCAATGTTC
ChIP m CD36 SITE1 R	GCCATCTAGAGCTAGGTTTCCA
ChIP m CD36 SITE2 F	TGTTGGCTATGAGGTTGGACA
ChIP m CD36 SITE2 R	TGCACATGAACATTGCATTCACTT
ChIP m CD36 SITE3 F	CCAATGGCAGGCACAATGTAAG
ChIP m CD36 SITE3 R	AAATCCACTAGCAGACAGGCAA

### Statistical analysis

The SPSS version 19 was used to carry out all statistical analyses. Data are depicted in terms of mean ± SD. Differences between the two groups were assessed via two-tailed Student’s *t* test. Differences between the three groups were assessed via ANOVA. Overall mice survival was determined with Kaplan–Meier analysis. All values of *P* < 0.05 were marked with an asterisk and were considered to be statistically significant.

## Supplementary information

Reproducibility checklist

SUPPLEMENTAL MATERIAL Figure S5-S6

SUPPLEMENTAL MATERIAL Figure S1-S4

SUPPLEMENTAL MATERIAL Figure Legends

Full scan lanes of WB

## Data Availability

All data generated or analyzed during this study are available from the corresponding author on reasonable request.
