# Supplementary figures and images for: B-cell lymphoma 6 alleviates nonalcoholic fatty liver disease in mice through suppression of fatty acid transporter CD36
Source: Cell Death Dis. 2022 Apr 18;13(4):359. doi: 10.1038/s41419-022-04812-x (PMC9016081; doi:10.1038/s41419-022-04812-x)

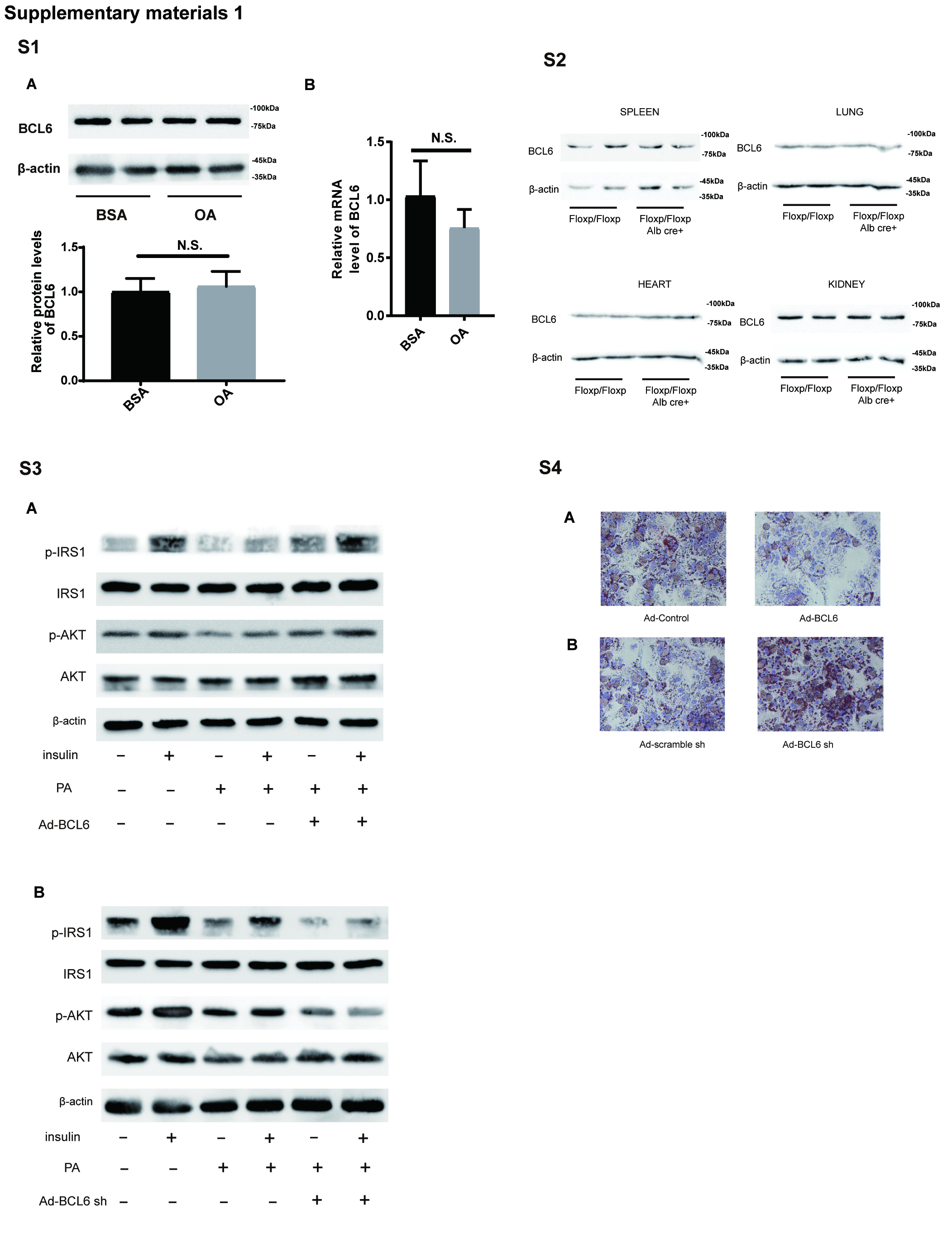

Supplement: Supplementary file 2 — SUPPLEMENTAL MATERIAL Figure S5-S6 [file 41419_2022_4812_MOESM2_ESM.tif]

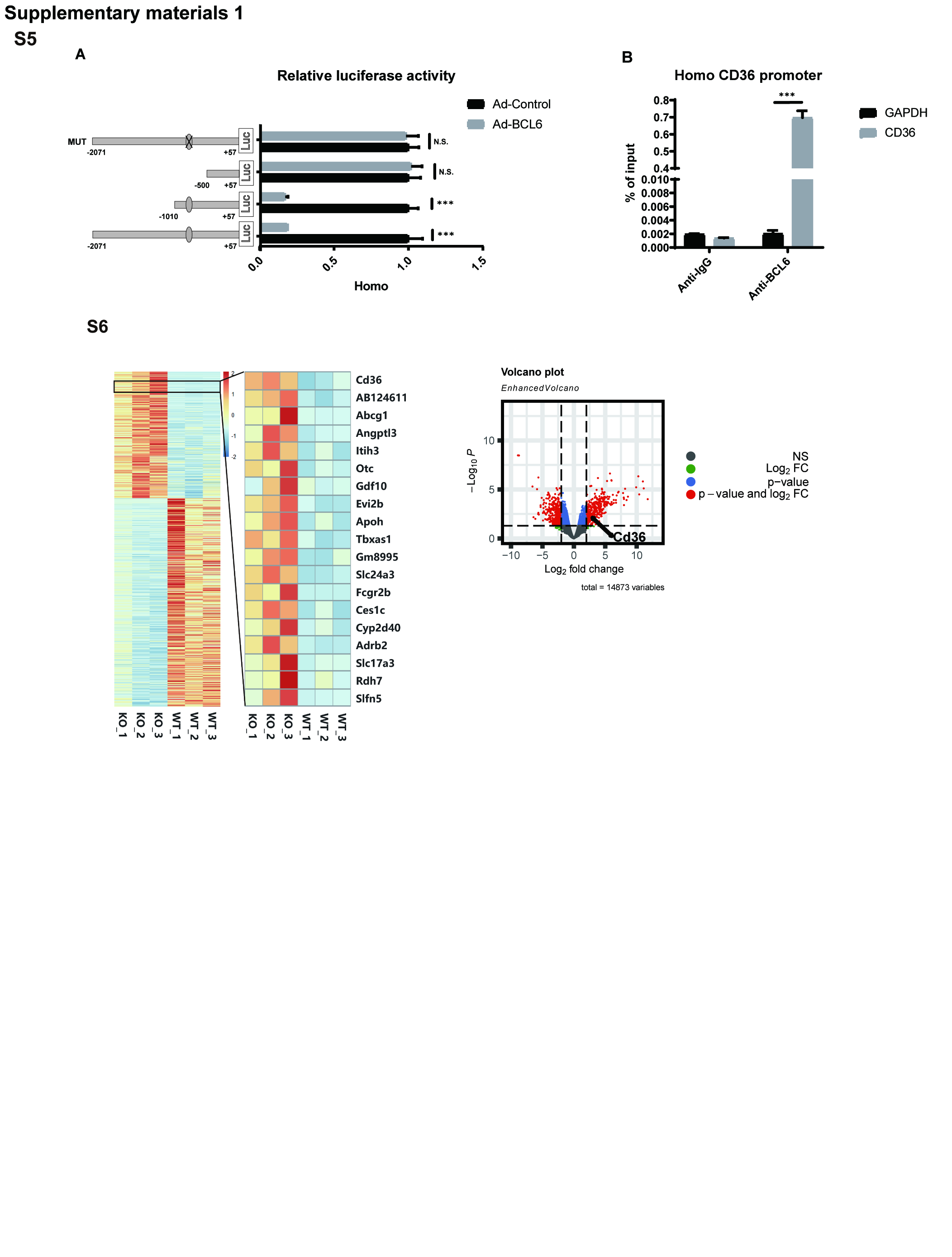

Supplement: Supplementary file 3 — SUPPLEMENTAL MATERIAL Figure S1-S4 [file 41419_2022_4812_MOESM3_ESM.tif]

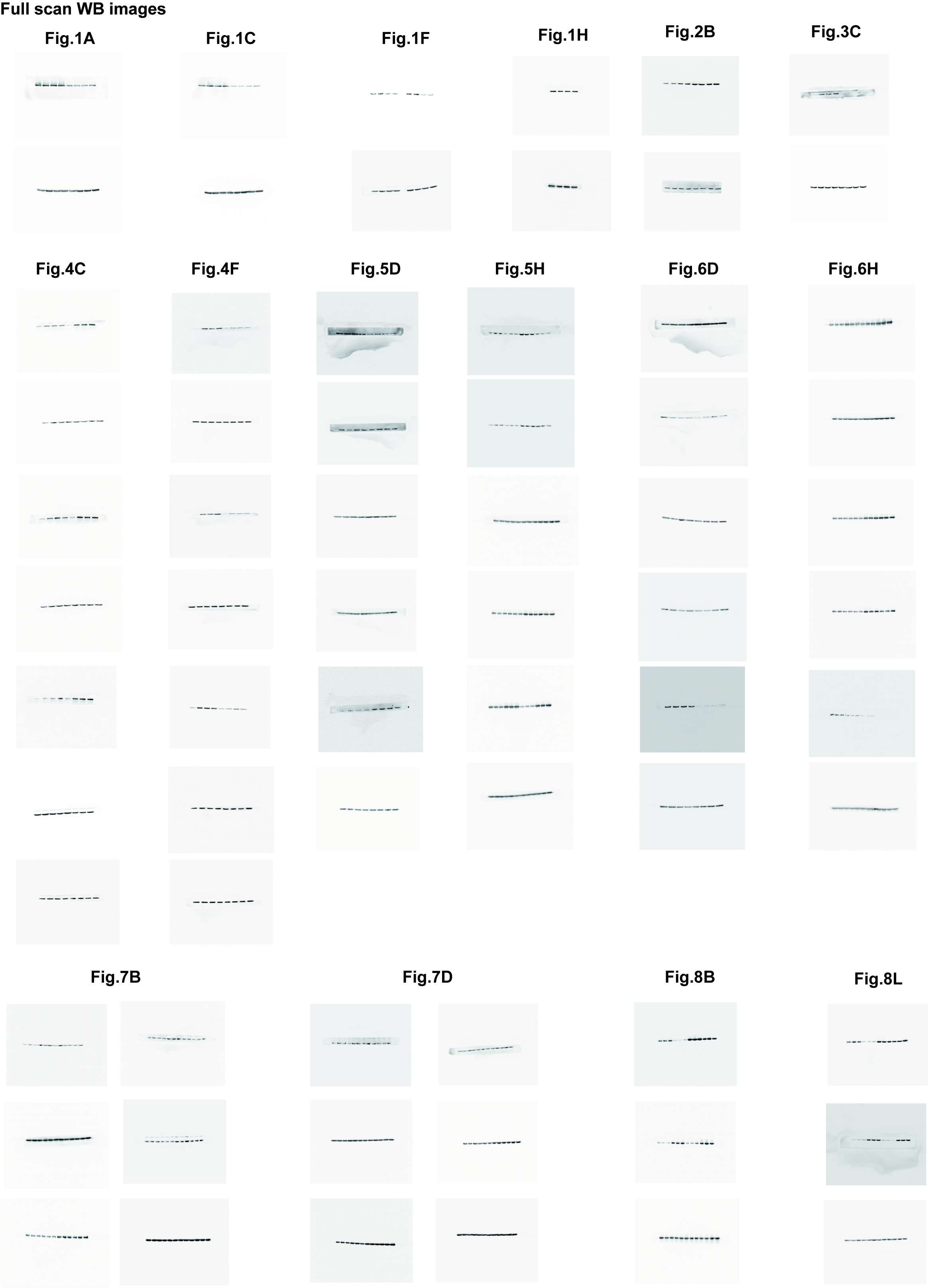

Supplement: Supplementary file 5 — Full scan lanes of WB [file 41419_2022_4812_MOESM5_ESM.tif]
