# Supplementary material for: B-cell lymphoma 6 alleviates nonalcoholic fatty liver disease in mice through suppression of fatty acid transporter CD36
Source: Cell Death Dis. 2022 Apr 18;13(4):359. doi: 10.1038/s41419-022-04812-x (PMC9016081; doi:10.1038/s41419-022-04812-x)
Supplement: Supplementary file 4 — SUPPLEMENTAL MATERIAL Figure Legends [file 41419_2022_4812_MOESM4_ESM.docx]

Supplementary materials Figure legends.

S1

(A) After isolated and stimulated with OA (0.3 mM) for 24 hours, BCL6 protein expression in primary hepatocytes was detected. (B) After isolated and stimulated with OA (0.3 mM) for 24 hours, BCL6 mRNA level in primary hepatocytes was detected. mRNA expression of target genes was normalized to that of β-actin. Data represent the mean ± SEM, *P < 0.05, **P < 0.01, ***P < 0.001 and N.S. indicates no significance between the two indicated groups.

S2

Protein level of BCL6 in other organs in BCL6-CKO mice

S3

(A) Representative western blot analysis (n = 3 western blots for each band) of phosphorylated (p-) and total IRS1, and AKT expression in the LO2 cells which respond to insulin or PA stimulation, upon manipulation of BCL6 expression via adenovirus containing BCL6 coding sequence (B) Representative western blot analysis (n = 3 western blots for each band) of phosphorylated (p-) and total IRS1, and AKT expression in the LO2 cells which respond to insulin or PA stimulation, upon manipulation of BCL6 expression via adenovirus containing BCL6 shRNA.

S4

Representative images of Oil red O staining of PA stimulated LO2 cells after BCL6 overexpression (A) and after BCL6 knockodown (B).

S5

(A) The human CD36 promoter was also studied to determine a possible BCL6 binding site, and the human CD36 promoter was constructed. BCL6 overexpression results in reduced luciferase activity of its reporter gene in the LO2 liver cell line. In JAPAR (http://jaspar.genereg.net/), we predicted that there was a possible binding site (-799) of BCL6 in the human CD36 promoter, and constructed reporter genes with different lengths and binding site mutations. Relative luciferase activity of CD36 promoters of different lengths was detected in LO2 cells after BCL6 overexpresion or not. (B) ChIP primers specific to the postulated binding site of BCL6 were created. Quantitative ChIP was performed in the LO2 cells using antibodies for BCL6 or IgG control to enrich for possible BCL6 binding site in the CD36 loci. The y axis represents fold enrichment of binding versus input, as compared with IgG control. Data represent the mean ± SEM, *P < 0.05, **P < 0.01, ***P < 0.001 and n.s. indicates no significance between the two indicated groups.

S6

Heat maps showing the changes in the expression of genes involved lipid metabolism in primary hepatocytes of BCL6-knockout and wild type mice. The color bar shows the gradient of the log2-fold changes in genes expression levels in the primary hepatocytes from BCL6-knockout mice relative to those in the primary hepatocytes from wild type mice. Thresholds used were p < 0.05 and a fold change > 1.5.
